# Brain strain: Blood flow and metabolism in environmental extremes

**DOI:** 10.1113/EP093359

**Published:** 2026-02-26

**Authors:** Dario Vrdoljak, Damian M. Bailey, Travis D. Gibbons, Philip N. Ainslie

**Affiliations:** ^1^ Centre for Heart, Lung and Vascular Health University of British Columbia, Okanagan Campus Kelowna British Columbia Canada; ^2^ Neurovascular Research Laboratory, Faculty of Life Sciences and Education University of South Wales Pontypridd UK; ^3^ Department of Biological Sciences Northern Arizona University Flagstaff Arizona USA

**Keywords:** apnoea, cerebral oxygen delivery, environmental stressors, exercise, high altitude, hyperthermia, hypothermia, hypoxia

## Abstract

This narrative review compares and contrasts the most commonly encountered environmental stressors on human cerebrovascular functioning. From high altitude and space, extreme apnoea, heat and cold stress, the impact of these stressors on the regulation of cerebral blood flow (CBF) and oxygen metabolism ($\mathrm{CM}R_{O_{2}}$) is discussed. As long as consciousness remains, $\mathrm{CM}R_{O_{2}}$ and oxygen delivery ($CD_{O_{2}}$) remain stable during acute and chronic exposure to poikilocapnic hypoxia. Such a response is possible with alterations in CBF to maintain a relatively stable $CD_{O_{2}}$. Likewise, the elevations in CBF during exercise in conditions of acute and chronic hypoxia seem to be appropriate to maintain stable $\mathrm{CM}R_{O_{2}}$. In freedivers, prolonged periods of apnoea during breath‐hold reflect marked hypoxaemia and acidosis. At these extremes in elite human freedivers, although elevations in CBF seem to maintain $CD_{O_{2}}$, there is evidence of reductions in $\mathrm{CM}R_{O_{2}}$. In contrast to hypoxia, heat‐ or cold‐induced hyperventilation and related hypocapnic‐induced vasoconstriction, marked reductions in CBF and $CD_{O_{2}}$ can occur. In the cold, however, the reductions in CBF seem to be partly compensated by elevations in blood pressure and haemoconcentration. In the heat, *Q*_10_‐mediated elevations in $\mathrm{CM}R_{O_{2}}$ are challenged by cerebral vasoconstriction and limited $CD_{O_{2}}$, especially when hyperventilation is pronounced. Furthermore, intracranial velocity seems stable during spaceflight despite elevations in $P_{\mathrm{aC}O_{2}}$. The implications of these changes in CBF and metabolism during environmental stressors are considered in the context of neuropsychological functioning. Finally, the limited research on cross‐exposures on cerebrovascular function is reviewed, and future research directions are proposed.

## Abstract (highlights)

- **What is the topic of this review?**
  This review examines the effect of the most commonly encountered environmental stressors, including high altitude and space, extreme apnoea, heat and cold stress, on human cerebral blood flow and metabolism.
- **What advances does it highlight?**
  When comparing the environmental stressors that may impact cerebral blood flow, alveolar ventilation‐induced changes in arterial $P_{CO_{2}}$ seems to be the most important (especially during heat and cold stress). During exposure to acute poikilocapnic hypoxia, the magnitude of the hypoxic ventilatory response determines the prevailing arterial $P_{CO_{2}}$ and degree of cerebral vasodilation. Understanding how the brain responds, adapts and tolerates these real‐world environmental stressors will inform preventative practices to manage potential neurological burden.

## 1 INTRODUCTION

The delivery of oxygen and nutrients to the brain by cerebral blood flow (CBF) is vital for supporting normal brain function, especially for providing stability in energy metabolism and related neural signalling. This is especially the case since, despite accounting for ∼2% of body mass, the brain utilizes a remarkable 20–30% of the total oxygen consumed by the body at rest (Kety & Schmidt, 1945; Scheinberg & Stead, 1949). Such high energy requirements of the brain make it susceptible to damage in conditions of insufficient oxygen supply. Theoretically, if blood flow and therefore oxygen delivery were abruptly stopped, carbohydrate available in tissue could fuel a normal metabolic rate for only a few minutes (reviewed in Rink & Khanna, 2011). However, such a scenario is highly unlikely, since such a state would lead to immediate unconsciousness before depletion of reserves owing to insufficient O_2_ availability (Smith et al., 2011; van Lieshout et al., 2003). The available O_2_ in tissue would be depleted in a few seconds at a normal oxygen metabolic rate, leading to an immediate compromise in neuronal activity (reviewed in Attwell & Laughlin, 2001; Attwell et al., 2010). Therefore, effective function of the vascular mechanisms that regulate cerebral oxygen delivery and oxygen extraction from capillary blood (termed the oxygen extraction fraction; OEF) is essential for continued cerebral oxidative metabolism ($\mathrm{CM}R_{O_{2}}$; e.g., Ainslie et al., 2014; Cohen et al., 1967; Kety & Schmidt, 1948). In order to satisfy the brain's O_2_ demand, the brain regulates cerebral oxygen delivery by modifying CBF according to arterial and capillary O_2_ availability; however, the oxygen extraction fraction may compensate as a last line of defence for preserving $\mathrm{CM}R_{O_{2}}$. As will be discussed later, although the capacity of the human brain to extract infinite O_2_ is limited, changes in OEF seem to be fundamentally influenced by CBF and its related cerebral oxygen delivery ($CD_{O_{2}}$) and energy demand. To adhere to the high metabolism of the brain, O_2_ delivery must be sufficient. Hence, the components of $CD_{O_{2}}$ are given in the following equations:

(1) $$CD_{O_{2}}=\left(\mathrm{CBF}\times C_{aO_{2}}\right)/100$$

(2) $$C_{aO_{2}}=\left(\left[\mathrm{Hb}\right]\times 1.36\times \%S_{aO_{2}}\right)/100+\left(0.003\times P_{aO_{2}}\right)$$

where [Hb] is the arterial haemoglobin concentration, 1.36 is the affinity of O_2_ for haemoglobin, 0.003 is the solubility of O_2_ dissolved in blood, and $P_{aO_{2}}$ is the partial pressure of O_2_ in arterial blood. Together, these variables yield a $CD_{O_{2}}$ of 10 mL O_2_ (100 g brain tissue)^−1^ min^−1^. Previously demonstrated typical values at sea level for Equations (1), (2), and (3), as given below, would be as follows: $S_{aO_{2}}$, 98%; Hb, 15.5 g (100 mL blood)^−1^; $P_{aO_{2}}$, 95 mmHg; CBF, 50 mL min^−1^ (100 g brain tissue)^−1^ (Ainslie et al., 2016; Lassen, 1985). Both metabolism and delivery of O_2_ depend upon blood flow, as demonstrated in Equations (1) and (3). As mentioned above, the main substrate that provides oxygen‐derived energy for the brain is glucose, which is delivered largely via the circulation; however, other substrates (e.g., lactate, ketones) can also be used for cerebral metabolism if available in the circulation.

Although there are many pathological conditions that are associated with reductions of brain oxygen supply (e.g., sleep apnoea and brain injury) (Hoiland et al., 2023b; Mohammadi et al., 2025), we herein focus on the impact of commonly encountered environmental stressors – hypoxia, thermal and gravity – on CBF regulation in the healthy human brain. We put particular focus on how physical activity might alter how these environments challenge cerebrovascular control, since survival in these contexts often requires physical exertion. First, we review the fundamental principles and current relationships that determine and influence CBF and O_2_ metabolism. Second, the impact of hypoxia is discussed with the objective of applying the fundamental principles of CBF regulation during (a) acute and chronic hypoxia, and extreme apnoea associated with freediving; (b) the influence and consequences of thermal strain on the human brain; and (c) cerebral blood flow regulation in microgravity. Third, the impact of combined environmental stressors on the brain is explored to provide important knowledge on real‐world environments, which always present as coexisting environmental challenges. Finally, future research directions are proposed, with particular emphasis on how the brain will be challenged by our rapidly changing climate.

### 1.1 Fundamental measures, principles and equations

To determine cerebral metabolism and delivery of O_2_, blood flow must be measured or estimated. The historical measurement of CBF in humans, both at rest and during exercise, has been reviewed extensively elsewhere (Tymko et al., 2018). In brief, although there is much merit in advanced neuroimaging approaches such as magnetic resonance imaging (MRI) and/or positron emission tomography (PET) for greater spatial resolution, these are not without limitations and fundamental assumptions and can typically only be performed during supine rest to avoid any movement artifact in a hospital setting. Typically, PET imaging would be considered the gold standard to measure regional blood flow and oxygen or glucose metabolism by tracking the uptake of injected radioactive tracers (usually H_2_^1^ ^5^O), whereas MRI can provide a non‐invasive index of flow in arteries of interest by tracking magnetically labelled blood water, contrast bolus dynamics or blood velocity. However, with few exceptions (Binks et al., 2008; Duffin et al., 2024; Harris et al., 2013; Zhang et al., 2020), MRI and PET have rarely been used to determine how environmental stressors impact CBF and its metabolism. The majority of studies have either incorporated measures of intracranial velocity via transcranial Doppler (TCD) or assessment of extracranial blood flow via Duplex ultrasound. Both approaches have advantages and disadvantages (reviewed in Hoiland & Ainslie, 2016; Thomas et al., 2015); the former measures intracranial *velocity* and the latter measures extracranial *flow*.

Cerebral metabolism is derived from the product of the rate of substrate delivery and the arterial–venous (a‐v) difference (i.e., extraction). As such, the way substrate extraction responds (or does not respond) to changes in delivery will determine the cerebral metabolic rate. Following the interconnection of a‐v O_2_ difference [arterial O_2_ content ($C_{aO_{2}}$) and venous O_2_ content (CvO_2_)] and delivery (CBF) is usually presented by the Fick equation (Fick, 1870), showcasing metabolism ($\mathrm{CM}R_{O_{2}}$):

(3) $$\mathrm{CM}R_{O_{2}}=\left(C_{aO_{2}}-C_{\bar{v}O_{2}}\right)\times \mathrm{CBF}$$

As mentioned above, both PET and MRI are rarely used in research examining the effects of environmental stressors, mainly because of the need for a supine resting position during measurement. Hence, when paired with measurement of CBF, arterial and internal jugular venous differences are viewed as the most applicable and gold‐standard method used in cerebrovascular research for relative environmental stress that may also involve movement or exercise (Cohen et al., 1967; Hoiland et al., 2023a; Kety, 1957); this approach can also readily quantify global oxygen, glucose and lactate metabolism (Dalsgaard et al., 2004; Koep et al., 2025; Rasmussen et al., 2010). The main disadvantages of this method are the invasive cannulation of a peripheral artery and internal jugular vein as well as the inability to determine regional oxygen metabolism.

As this review deals with the main environmental stressors that are affecting cerebral vasculature, it is important to demonstrate the relationship between temperature and chemical/biological reactions. Svante Arrhenius quantified this relationship and created the *Q*_10_ temperature coefficient, which describes the rate of change in any biological or chemical system from a change in temperature of 10°C (Mundim et al., 2020), expressed as:

(4) $$Q_{10}={\left[\frac{k\left(T_{2}\right)}{k\left(T_{1}\right)}\right]}^{10/\left(T_{2}-T_{1}\right)}$$

Where *T* is the temperature in degrees, and *k* is the rate constant that describes the rate of biological process (i.e., metabolic rate). In mammalian biological tissues, the *Q*_10_ is typically between 2 and 3 (Sakoh & Gjedde, 2003), with a value of ∼2.2–2.4 generally associated with the brain (McCullough et al., 1999). By way of this principle, a nonlinear reduction or increase in metabolism is associated with progressive hypothermia or hyperthermia, respectively. For example, a reduction in core temperature from 38 to 35°C is predicted to reduce metabolism by ∼25%, or by ∼5%‐7% per degree Celsius (Sakoh & Gjedde, 2003). In terms of hyperthermia, elevations in core temperature from 38 to 42°C is predicted to increase metabolism by ∼45% or by ∼10–20% per degree Celsius increase in core temperature (Bain et al., 2014; Bennett, 1984; Donnelly et al., 1956).

## 2 CBF REGULATION

**FIGURE 1:**
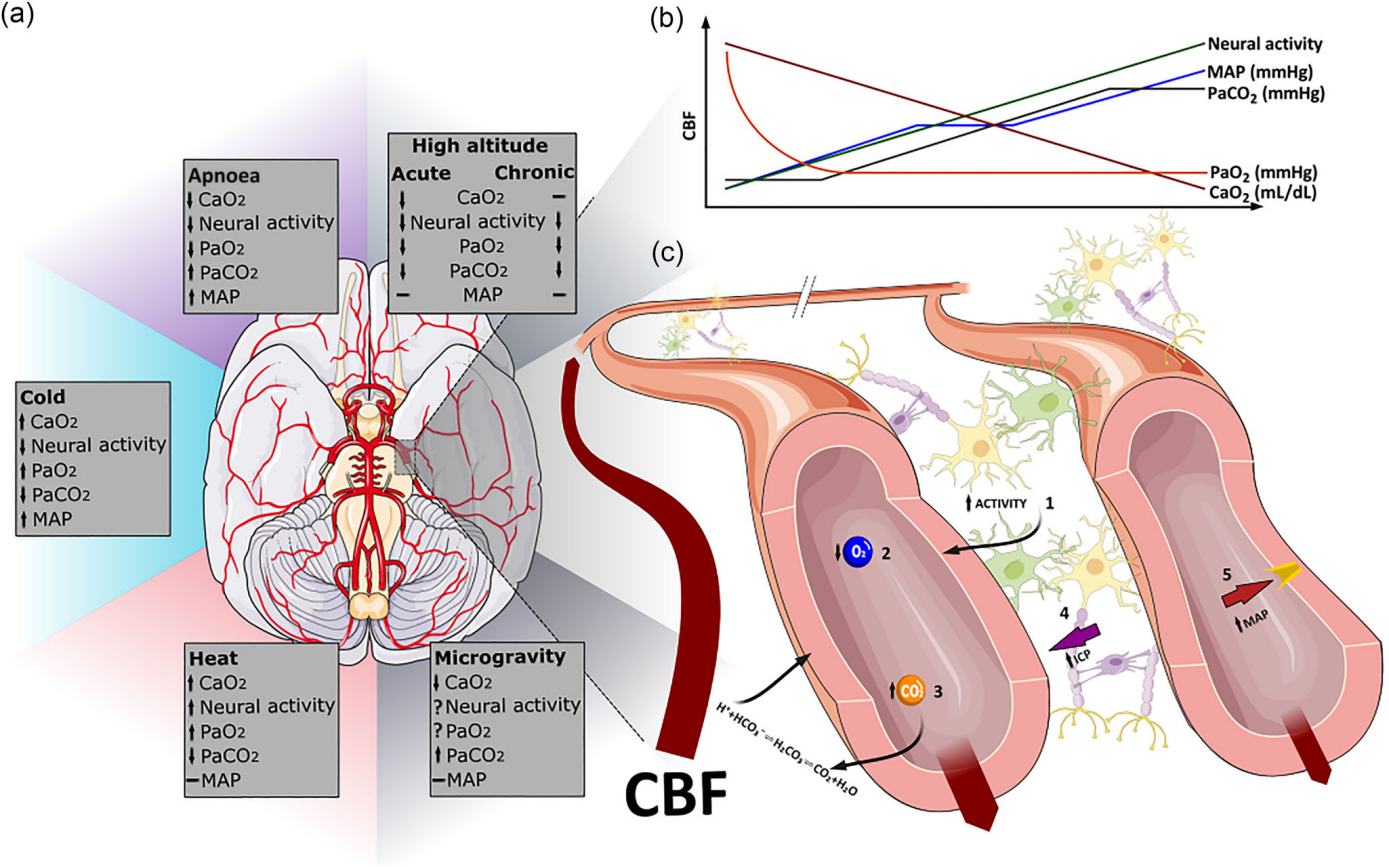
Mechanisms of cerebral blood flow regulation at rest during exposure to selected environmental stressors. (a) Impact of different environmental stressors ($C_{aO_{2}}$, metabolic pathway, $P_{aO_{2}}$, $P_{\mathrm{aC}O_{2}}$, cerebral SNA, blood pressure) on cerebrovascular function. (b) The CBF autoregulation in regard to different physiological changes and the response during different environmental stressors (denoted with arrows). (c) The cerebral vasculature response to given stressors: the interbrain functions connected to cerebral autoregulation are numbered as follows: (1) neurovascular coupling: neuronal activity is transmitted through astrocytes to the vascular wall, mediating vasodilation; (2) hypoxia‐mediated vasodilation: reduction in the partial pressure of oxygen ($P_{aO_{2}}$) triggers a cascade of processes and inducing dilation of cerebral vessels; (3) cerebrovascular reactivity to changes in $P_{\mathrm{aC}O_{2}}$: increases in $P_{\mathrm{aC}O_{2}}$ mediate changes in extracellular pH enabling relaxation whereas reductions in $P_{\mathrm{aC}O_{2}}$ mediate vasoconstriction; (4 and 5) cerebral autoregulation (CA) and autonomic control: CA is a critical mechanism triggering changes in cerebrovascular resistance in response to changes in system blood pressure to maintain CBF in an attempt to prevent over‐ or under perfusion. Although controversial and unclear in humans, there is likely some autonomic control of the cerebrovasculature that aids in the management of CA (especially in the prevention of overperfusion). Finally, it should be noted that (1)–(5) rarely occur in isolation in the otherwise healthy human. For example, changes in arterial blood gases [(2) and (3)] may profoundly influence mean arterial pressure (MAP) and intracranial pressure (ICP); changes in MAP may markedly alter the capacity of the brain to vasodilate or constrict during changes in arterial blood gases; and changes in neural activity may also modify the capacity of the brain to vasodilate or constrict. For detail reviews on these topics, see Bergh & Ekblom (1979), Caldwell et al. (2024), Claassen et al. (2021), Hoiland et al. (2019), Lefferts et al. (2020), Parkin et al. (1999), Phillips et al. (2016), Tzeng & Ainslie (2014), Willie et al. (2014).

The brain is dependent upon constant oxygen and nutrient delivery to sustain homeostatic functions. As highlighted in Equation (2), $CD_{O_{2}}$ is directly proportional to CBF and arterial oxygen content ($C_{aO_{2}}$). CBF is directly related to cerebral perfusion pressure and inversely proportional to cerebrovascular resistance. The regulation of CBF is complex, heterogeneously distributed across brain regions, and governed by multiple complex and often redundant pathways (reviewed by Willie et al., 2014). These main pathways are illustrated in Figure 1, and include neurovascular coupling, arterial blood gas influences on vasomotor tone, cerebral autoregulation, and autonomic control. Although summarized in Figure 1, it is beyond the scope of this short review to detail these fundamental mechanisms that regulate CBF. Rather, brief overview of each of these processes is provided and the interested reader is referred to detailed reviews on this topic (Claassen et al., 2021; Willie et al., 2014).

### 2.1 Neurovascular coupling

The matching of cerebral metabolism with brain perfusion is referred to as neurovascular coupling. Activation of distinct anatomical foci in the brain results in a regional increase in CBF to meet its metabolic demands. The importance of neurovascular coupling (NVC) is to ensure CBF is finely tuned to neuronal activity, which is essential for maintaining optimal brain function (Yang et al., 2024). Two general mechanisms have been proposed that determine NVC. First, a ‘feedback system’, where changes in O_2_, CO_2_, pH and other vasoactive substances drive local vasodilation and increase CBF in some brain regions (Iadecola, 2017). Such increases occur following neural activation and can exceed metabolic demands even under conditions of an abundance of glucose and O_2_ (Attwell et al., 2010; Fox & Raichle, 1986; Raichle & Mintun, 2006). Second, since increases in CBF occur rapidly and in excess of the metabolic demand, a ‘feedforward’ theory explains that coupling takes place in the neurovascular unit (between neurons, glia and the microvasculature) (Hoiland et al., 2020; Iadecola, 2017; O'Gallagher et al., 2022). It seems likely, however, that the NVC responses involve a combination of both ‘feedback’ and ‘feedforward’ mechanisms (Iadecola, 2017). Regardless of the contribution of these mechanisms, NVC needs to be maintained so that glucose delivery and metabolism are maintained in response to neural activation. Hence, NVC is a critical component of CBF regulation, having implications for cerebrovascular, autonomic, and cognitive dysfunction (reviewed in Claassen et al., 2021; Phillips et al., 2016).

### 2.2 Arterial blood gases

Changes in arterial blood gases, specifically carbon dioxide ($P_{\mathrm{aC}O_{2}}$) and oxygen ($P_{aO_{2}}$), trigger changes in vascular tone and CBF (Kety & Schmidt, 1948; Wasserman & Patterson, 1961). Even small (1–2 mmHg) changes in $P_{\mathrm{aC}O_{2}}$ alter extracellular pH by enabling movement of CO_2_ across the blood–brain barrier. When this occurs, the altered pH in the extracellular fluid stimulates concomitant changes in the vasomotor tone of the cerebral vessels, thus altering CBF. In contrast to changes in $P_{\mathrm{aC}O_{2}}$, the cerebrovasculature is relatively insensitive to arterial hypoxaemia. For example, it is generally not until $P_{aO_{2}}$ is reduced below ∼50 mmHg that vascular tone is reduced and vasodilation occurs (referred to as hypoxia‐mediated vasodilation). As reviewed in depth elsewhere (Carr et al., 2024), this hypoxia‐mediated vasodilation elevates CBF and acts to maintain $CD_{O_{2}}$. Note that CBF is also influenced by reductions in $C_{aO_{2}}$ per se (Gottesman et al., 2012; Hare, 2004; Hoiland et al., 2023a; Paulson et al., 1973; Todd et al., 1994), indicating that reductions in $P_{aO_{2}}$ per se are not obligatory in these pathways.

### 2.3 Autonomic control and cerebral autoregulation

Cerebral perfusion pressure is largely determined by the difference of mean arterial pressure (MAP) with intracranial pressure (ICP); thus, changes in MAP directly influence cerebral perfusion pressure, altering CBF. Cerebral autoregulation (CA) mechanisms trigger changes in cerebrovascular resistance in response to changes in system blood pressure to maintain CBF and $CD_{O_{2}}$ demands (Tzeng & Ainslie, 2014). This encompasses both acute, transient changes (termed ‘dynamic’ CA) and slower, sustained pressure changes (termed ‘static’ CA). The final and related, albeit subtle, regulator of both CBF and CA is the autonomic nervous system (reviewed in Brassard et al., 2017; Koep et al., 2022). Autonomic control of the cerebrovasculature is complex and dependent on various factors such as receptor type and density, neurotransmitter concentration and vessel location to maintain adequate perfusion. Sympathetic activation results in the release of neurotransmitters to act upon the vasculature through adrenergic receptors but can have conflicting actions of dilation or constriction (for reviews, see: Brassard et al., 2017; Koep et al., 2022).

The following sections, the direct and indirect effects of hypoxia, temperature and microgravity that can influence the main factors that regulate CBF are summarized and compared.

## 3 INFLUENCE OF ACUTE AND CHRONIC HYPOXIA ON CBF REGULATION

### 3.1 Acute hypoxia

**FIGURE 2:**
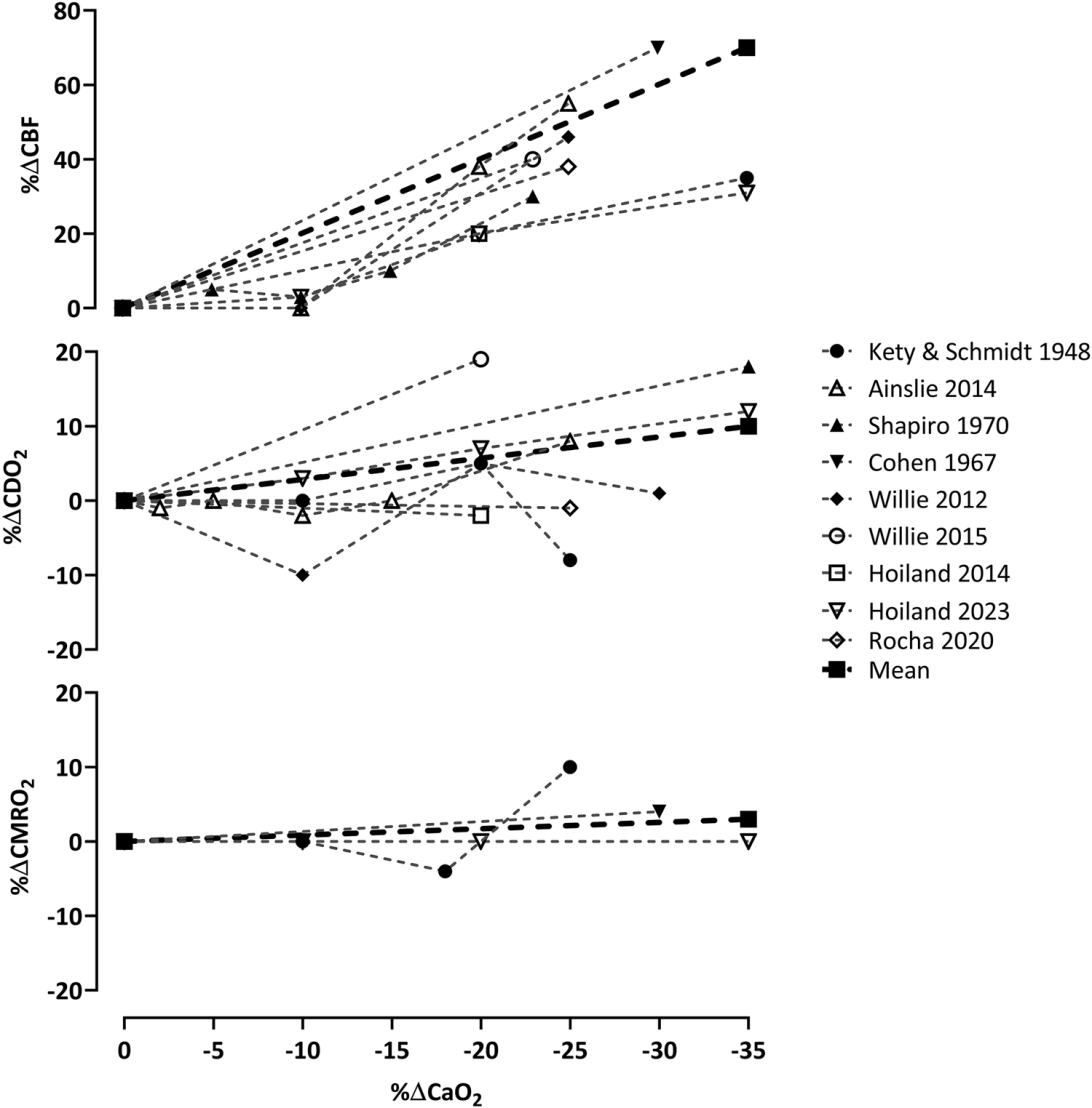
Cerebral blood flow (CBF) and oxygen delivery ($CD_{O_{2}}$) during acute hypoxemic hypoxia in humans. Each line represents a published study, with the bold line indicating the weighted group mean. As $C_{aO_{2}}$ is reduced, $CD_{O_{2}}$ and CBF increase compared to baseline, which is shown as a percentage change. However, $\mathrm{CM}R_{O_{2}}$ shows little change with reductions in $C_{aO_{2}}$. The studies included in the figure are Ainslie & Subudhi (2014), Cohen et al. (1967), Hoiland et al. (2023a), Kety & Schmidt (1948), Rocha et al. (2020), Shapiro et al. (1970), Willie et al. (2015a, 2012).

Acute hypoxia refers to a sudden and significant reduction in the availability of oxygen to tissues, occurring over a short period (seconds to minutes to hours). In addition to laboratory based experimental situations, acute hypoxia can also occur during rapid depressurization of aircraft, sudden ascent to high altitude or during prolonged apnoea. During these low O_2_ periods, $CD_{O_{2}}$ is generally maintained via an increase in CBF (Hoiland et al., 2016; Willie et al., 2015a) to prevent metabolic compromise and/or structural damage to the neurovascular unit (reviewed in Ainslie & Duffin, 2009; Attwell et al., 2010) (see Figures 2 and 4). On the other hand, the effects of acute hypoxia on $\mathrm{CM}R_{O_{2}}$ seem minimal, with reports of slight (5–8.5%) increases (Vestergaard et al., 2016; Xu et al., 2012) or no changes (Ainslie et al., 2014; Cohen et al., 1967; Kety & Schmidt, 1948). Regardless of the stability in global $\mathrm{CM}R_{O_{2}}$, extramitochondrial cellular processes are sensitive to small hypoxic changes, even when $CD_{O_{2}}$ is sufficient to maintain bioenergetic function (Pulsinelli, 1985). Synthesis of enzymes and related neurotransmitters, as well as neurotransmitter dysfunction, likely also occur during acute or chronic reductions in $P_{aO_{2}}$ (Ainslie et al., 2016; Hornbein, 2001; Kumar, 2011). However, although now potentially feasible with metabolomic or proteomic approaches, such molecular changes have not been examined in humans. Additionally, although detrimental effects on central nervous system (CNS)‐impaired cognitive function may appear following hypoxia (Ando et al., 2020; McMorris et al., 2017; Taylor et al., 2016; Virués‐Ortega et al., 2004; Wilson et al., 2011), this is not a universal finding (Fugate et al., 2013; Noble et al., 1993; Sun et al., 2019; Zhang et al., 2025).

### 3.2 Chronic hypoxia

**FIGURE 3:**
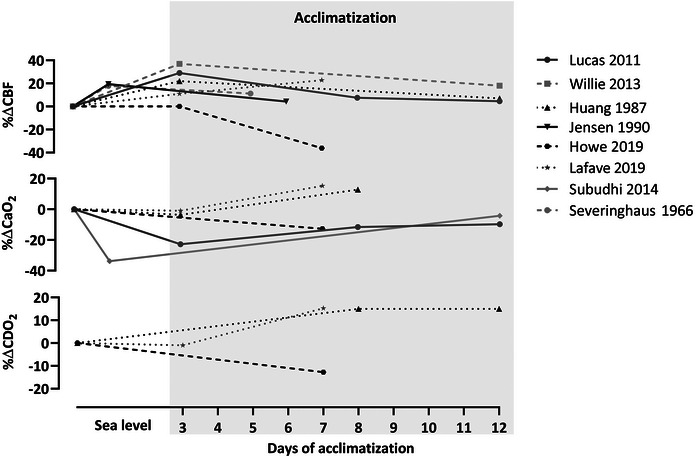
Schematic diagram presenting typical changes in cerebral blood flow (CBF), cerebral oxygen delivery ($CD_{O_{2}}$), and the arterial oxygen content ($C_{aO_{2}}$ at arrival to high altitude and followed by acclimatization period. At arrival CBF increases from baseline resting values comparative to $C_{aO_{2}}$, to maintain stable $CD_{O_{2}}$. Throughout the acclimatization period, both CBF and $C_{aO_{2}}$ return to baseline values and, therefore, $CD_{O_{2}}$ is once again maintained. Data points are presented from Howe et al. (2019), Huang et al. (1987), Jensen et al. (1990), Lafave et al. (2019), Lucas et al. (2011), Severinghaus et al. (1966), Subudhi et al. (2014), Willie et al. (2014). Mean responses are presented as a solid line indicating the line of best fit through the amalgamated data. Data during ascent and acclimatization are presented as percent change from sea level or near sea level values as reported in each respective study against altitude (ascent) or duration of stay (acclimatization). Arterial oxygen content was determined directly from arterial blood sampling or indirectly through measurement of peripheral oxygen saturation and haemoglobin or hematocrit. Cerebral oxygen delivery was determined by multiplying the calculated oxygen content by the respective measure of cerebral blood flow (see Equation (2) above) utilized in each respective study (i.e., duplex ultrasound, transcranial doppler ultrasound, or Fick method).

**FIGURE 4:**
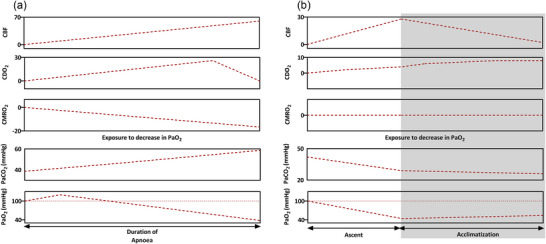
Integrative model of cerebrovascular comparing the changes CBF, $CD_{O_{2}}$, and $\mathrm{CM}R_{O_{2}}$ to apnoea (a) and high altitude (b) during ascent and with initial acclimatization to approx. 5000 m. Apnoeic stress influences more marked changes in CBF, oxygen delivery and metabolism in cerebral vasculature than altitude hypoxia, since there is greater arterial hypoxaemia and acidosis in the absence of acclimatization. $CD_{O_{2}}$ and $\mathrm{CM}R_{O_{2}}$ are reduced in the latter part of apnoea whereas CBF increases until apnoea cessation. On the other hand, during initial ascent to high altitude, $CD_{O_{2}}$ and $\mathrm{CM}R_{O_{2}}$ seem to be maintained via appropriate elevations in CBF. During acclimatization, CBF returns towards sea level values upon restoration of $C_{aO_{2}}$ and $CD_{O_{2}}$ and $\mathrm{CM}R_{O_{2}}$ also remain stable (see Figure 3). Redrawn and modified from Ainslie & Subudhi (2014), Andersson & Schagatay (1998), Bain et al. (2016a), Baumgartner (2003), Willie et al. (2015a).

During the acute stages of high‐altitude exposure, the reduced barometric pressure results in reductions in $P_{aO_{2}}$ and arterial oxygen saturation ($S_{aO_{2}}$), leading to overall reductions in arterial oxygen content $C_{aO_{2}}$. This necessitates a compensatory increase in CBF. However, across multiple studies using both rapid vehicular and gradual ambulatory ascent methodologies, $CD_{O_{2}}$ appears to be largely preserved across the varied hypoxic stimuli experienced at high altitude (Figures 3 and 4). In the initial stages of high‐altitude exposure, increases in alveolar ventilation result in a marked reduction in $P_{\mathrm{aC}O_{2}}$ and small elevations in $P_{aO_{2}}$. Metabolic compensation then acts to compensate for this respiratory alkalosis in an attempt to normalize pH. Therefore, the cerebrovasculature is undergoing both stimulation (from the hypoxaemia) and inhibition (from the alkalosis) under these conditions (reviewed in Hoiland et al., 2018). Moreover, with acclimatization, $C_{aO_{2}}$ is normalized via the persistent hyperventilation and elevations in haemoglobin levels. Despite these complex and conflicting influences on cerebrovascular tone, multiple studies have shown that CBF at high altitude appears to be modulated in a manner such that $CD_{O_{2}}$ is maintained, overcoming the countering influence of hypocapnia, with $C_{aO_{2}}$ being the principal determinant of CBF as shown in Figure 3. Despite these temporal changes in CBF, $\mathrm{CM}R_{O_{2}}$ seems to be stable at altitudes between 3800 and 5300 m owing to the tight regulation of $CD_{O_{2}}$ (Møller et al., 2002; Severinghaus et al., 1966; Willie et al., 2015b). In summary, the initial increase in CBF at high altitude seems to be explained by a reduction in $C_{aO_{2}}$ as well as haemoconcentration and ventilatory acclimatization that act to normalize CBF and maintain a stable $CD_{O_{2}}$ (Howe et al., 2019; Subudhi et al., 2010).

### 3.3 What happens to the brain during physical activity in hypoxic environments?

The CBF response to exercise in a normoxic environment is usually influenced by neural activity/motor demands and alveolar ventilation‐induced alterations in $P_{\mathrm{aC}O_{2}}$ (LaManna et al., 1993; Lambertsen et al., 1959; Rasmussen et al., 2010). To a lesser extent, depending on the type of exercise, MAP and cardiac output may also influence the CBF pattern during exercise (Ogoh et al., 2005; Smith et al., 2016). Nevertheless, following acclimatization to high altitude (5000 to 5400 m), studies report that exercise induces an appropriate increase in CBF and haemoconcentration, which also act to maintain $CD_{O_{2}}$ and $\mathrm{CM}R_{O_{2}}$ at levels similar to that observed during sea level exercise (Møller et al., 2002; Smith et al., 2014). Increases in core (and thus brain; Nybo et al., 2002b) temperature may also play a role in increasing CBF during physical exercise (Caldwell et al., 2020), and data from our lab indicate that core temperature changes during matched work rate exercise are similar at sea level and after acclimatization to high‐altitude.

### 3.4 CBF regulation during extreme apnoea

**FIGURE 5:**
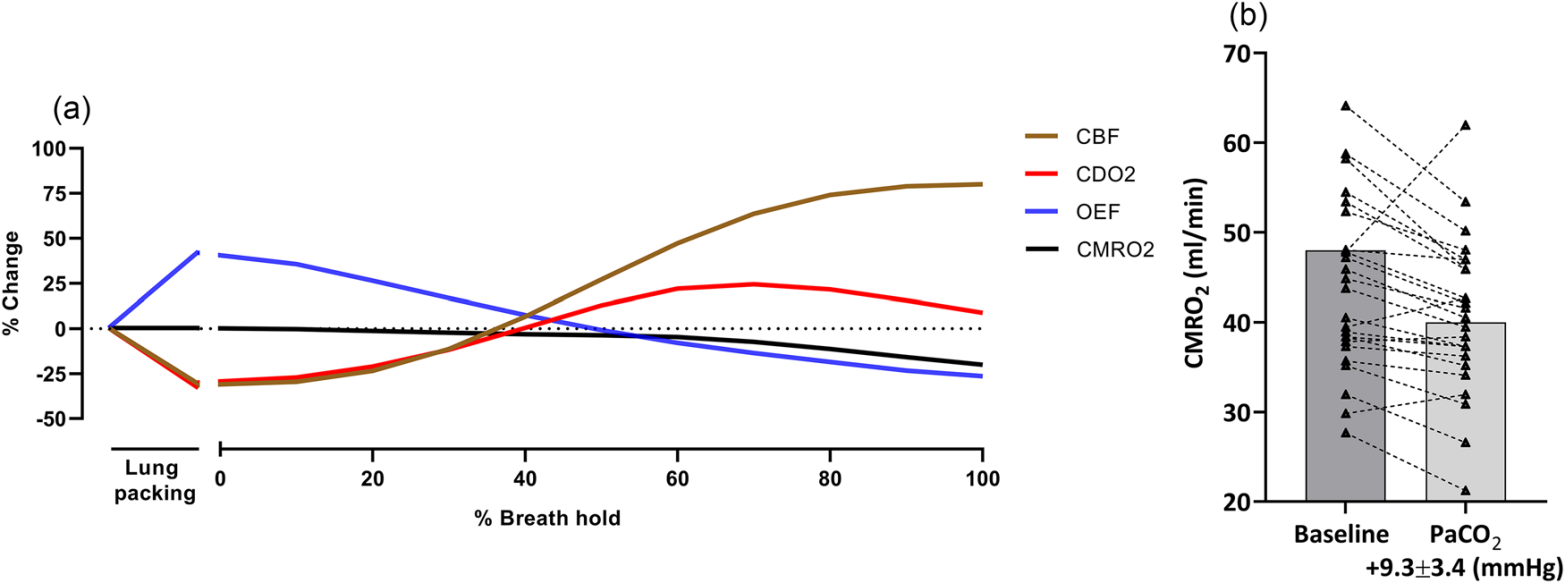
(a) Schematic diagram presenting typical percentage changes in cerebral blood flow (CBF; brown), cerebral oxygen delivery ($CD_{O_{2}}$; red), cerebral oxygen extraction (OEF; blue) and the cerebral metabolic rate of oxygen ($\mathrm{CM}R_{O_{2}}$; black) throughout maximal dry breath holds of ∼5 min or longer in elite apnoeists, performed with glossopharyngeal insufflation (lung packing). (b) The $\mathrm{CM}R_{O_{2}}$ is mediated by an increase in $P_{\mathrm{aC}O_{2}}$. At the initial stages of the breath hold, CBF and $CD_{O_{2}}$ are depressed below baseline resting values, primarily attributable to the hypotension and hypocapnia subsequent to glossopharyngeal insufflation. The reduction of $\mathrm{CM}R_{O_{2}}$ at the latter end of the breath hold is primarily attributed to the hypercapnia. Modified and redrawn from Bain et al. (2017, 2016a, 2015a), Caldwell et al. (2024), Willie et al. (2015a).

Apnoea is defined as complete cessation of breathing and is reflected in marked arterial hypoxaemia and hypercapnia (Lindholm & Lundgren, 2006). To maintain consciousness, the human body slows systemic oxidative metabolism to delay critical levels of hypoxaemia and prolongs the apnoea breaking point in elite divers (Bain et al., 2015a). With this adaptation, human apnoea times reached values of more than 10 min (officially recognized world record in static apnoea: 11:35 min). During such extreme apnoeas, $P_{aO_{2}}$ drops to values as low as ∼30 mmHg, whereas $P_{\mathrm{aC}O_{2}}$ rises to ∼60 mmHg at the break point (Lindholm & Lundgren, 2006; Willie et al., 2015a). Such high hypoxaemia and hypercapnia induce cerebrovascular dilation in combination with hypertension and the subsequent increases in perfusion pressure (Bain et al., 2016b, 2015b; Cross et al., 2013; Stembridge et al., 2017; Willie et al., 2015b), leading to an increase in CBF and maintained $CD_{O_{2}}$ (see Figure 5).

The elevation in CBF also needs to compete with other attenuating factors, including profound sympathoexcitation and increased ICP. In spite of a hypothetical increase in ICP, previous studies included indirect measures of ICP that were estimated via measures of internal jugular venous pressure (Stembridge et al., 2017) and subarachnoid space width (Winklewski et al., 2015). Here, it was estimated that ICP increases during the last quarter of a maximal breath hold coincided with surges in systolic pressure/MAP, which induced a decrease in cerebral perfusion pressure (Bailey et al., 2022; Stembridge et al., 2017). Reports indicate that $\mathrm{CM}R_{O_{2}}$ remains unaltered until the last 20% of a prolonged breath hold (see Figure 5a; Bain et al., 2017, 2016a). During the final stages of apnoea, however, $\mathrm{CM}R_{O_{2}}$ is reduced by ∼25% (from ∼48 to 36 mL min^−1^; Bain et al., 2017, 2016a). Follow‐up studies revealed that hypercapnia rather than hypoxia acts to reduce $\mathrm{CM}R_{O_{2}}$ during extreme apnoea (Bain et al., 2017, 2016a, 2018a; Bain et al., 2018b). Similarly, hypercapnia per se independent of apnoea (i.e., during normal breathing) has recently been reported to attenuate $\mathrm{CM}R_{O_{2}}$ in otherwise healthy humans (Caldwell et al., 2024; Figure 5b). The direct and indirect mechanisms by which CO_2_ might influence $\mathrm{CM}R_{O_{2}}$ remain to be established.

### 3.5 Implications on cerebrovascular function in hypoxia

The physiological perturbations resulting from hypoxia extend beyond metabolic adjustments; these perturbations can lead to temporary or permanent cognitive impairment (Phillips et al., 2016; Turner et al., 2015; Uchida et al., 2020a, 2020b), altered neurophysiological states (Binks et al., 2008; Harris et al., 2013; Lawley et al., 2017a) and potential neurodegeneration (Chand Dakal et al., 2024; Yuan et al., 2023; Zhang & Le, 2010) if oxygen deprivation persists. However, an adverse change in neurophysiological outcomes in such pathologies is not a universal finding (Fugate et al., 2013; Noble et al., 1993; Sun et al., 2019; Zhang et al., 2025). Regardless, understanding how the brain adapts to hypoxia is critical for understanding brain health and developing targeted interventions for hypoxia‐related conditions such as high‐altitude military aviation and related neurological‐related pathologies. Cerebrovascular regulation during breath holding is complicated through an integration of multiple factors, since oxygen deprivation occurs rapidly and intermittently rather than chronically. Some studies show that these acute hypoxic episodes, especially when repeated over time, can lead to transient cognitive deficits (Fan et al., 2024), altered cortical excitability and neural function (Steinberg et al., 2017), and in some cases may contribute to long‐term neural consequences such as oxidative stress, neuronal injury or neurodegeneration (reviewed in Ribeiro et al., 2024). This topic is still being explored with some studies showing no effect of a prolonged breath‐hold diving session (up to 300 s) (Ratmanova et al., 2016) or lifelong exposure to breath‐hold diving (~20 years) (Ridgway & McFarland, 2006) on impairments in cerebrovascular function. Nevertheless, such insights are critical for improving safety, optimizing performance and mitigating potential neurological consequences in freedivers and others exposed to intermittent hypoxic stress.

## 4 INFLUENCE OF TEMPERATURE ON CBF REGULATION

### 4.1 Hyperthermia

**FIGURE 6:**
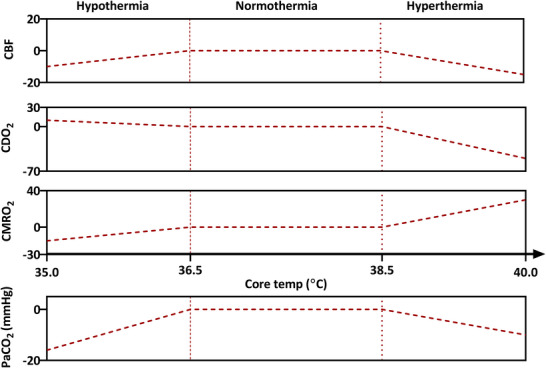
Integrative model of cerebrovascular changes to progressive passive cold and heat stress. In the healthy human, increases in core temperature of less than 1°C generally proffer little challenge to the brain. Thereafter, hyperthermic‐induced hyperventilation reduces the arterial partial pressure of CO_2_ ($P_{\mathrm{aC}O_{2}}$), causing cerebral vasoconstriction and reductions in cerebral blood flow (CBF). The cerebral metabolic rate of oxygen ($\mathrm{CM}R_{O_{2}}$) progressively increases (with heat) or decreases (with cold), attributed to the inherent biological response to temperature changes (*Q*_10_ effect). Redrawn and modified from Bain et al. (2015b), Gibbons et al. (2021), Howe et al. (2025).

Although on a continuum, mild, moderate and severe heat stress are defined as a core temperature increase of ≤1.0, 1.0–1.5, and ≥1.5°C, respectively (Bain et al., 2015b; Nielsen & Nybo, 2003). Changes in core temperature can be induced by passive or exertional means, depending on the source; as such, the cerebrovascular responses differ (Gibbons et al., 2021). It is initially estimated that each degree Celsius increase in core temperature during supine passive heat stress resulted in a reduction in CBF by ∼10–15% (Bain et al., 2015b, 2013; Ogoh et al., 2014) (see Figure 6). However, recent findings show that CBF (when measured directly) does not change during passive heating until core temperature is >1.5°C above baseline (Bain et al., 2020; Caldwell et al., 2020). At this threshold, although variable within and between individuals, heat stress‐induced hyperventilation appears to drive reductions in CBF. Furthermore, reductions in mean arterial pressure and potential increases in intracranial pressure (Gibbons et al., 2021) may augment hypocapnia‐mediated reductions in CBF during severe heat strain via reduced cerebral perfusion pressure. Cerebral vasoconstriction from increases in sympathetic nerve activity (Brothers et al., 2009) has also been proposed but not supported in other studies (Bain et al., 2013; Gibbons et al., 2021). It seems, therefore, that by far the most powerful influence is heat‐induced hyperventilation and related hypocapnic‐induced vasoconstriction, leading to marked reductions in CBF and $CD_{O_{2}}$ (Bain et al., 2014; Gibbons et al., 2021).

**FIGURE 7:**
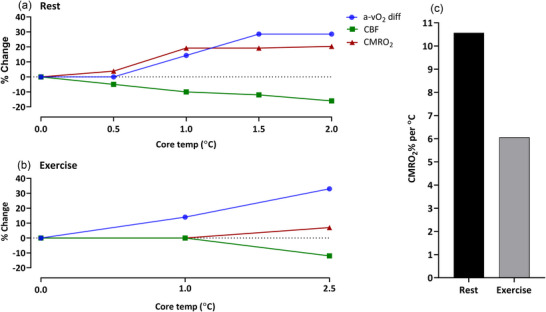
(a, b) Schematic diagram presenting typical percentage changes in cerebral blood flow (CBF; green), arterial–venous oxygen content difference (a‐vO_2_ diff; blue) and the cerebral metabolic rate of oxygen ($\mathrm{CM}R_{O_{2}}$; red) throughout increments of heating at rest (a) and exercise (b). (c) The percentage increase in $\mathrm{CM}R_{O_{2}}$ per °C elevation in *T*_core_ is shown for both rest and exercise. The increase in core temperature influenced a slight decrease in CBF accompanied by an increase in both $\mathrm{CM}R_{O_{2}}$ and a‐vO_2_ difference. Modified and redrawn from Bain et al. (2020), Nybo et al. (2002a).

During passive hyperthermia, whole body metabolic rate increases by ∼25% for a 1.5–2°C rise (Saxton, 1981), which is influenced, in part, by the Arrhenius activation law (or *Q*_10_, temperature coefficient). The *Q*_10_ implies that a rise in 2°C from 37°C should yield an increase in metabolic rate of ∼10% (South, 1958). Previously, the $\mathrm{CM}R_{O_{2}}$ and *Q*_10_ interconnection was demonstrated only in anaesthetized‐animal studies. They show that for every 1°C rise in core temperature, $\mathrm{CM}R_{O_{2}}$ increases by 5–10% (Busija et al., 1988; Carlsson et al., 1976; Nemoto & Frankel, 1970a, 1970b). In humans, PET measurements during passive heating yielded increased metabolic rate of glucose in some (hypothalamus, thalamus, corpus callosum, cingulate gyrus and cerebellum) but not all (caudate, putamen, insula and posterior cingulum) regions of the human brain (Nunneley et al., 2002). Also in humans, Bain et al. (2020), showed that global $\mathrm{CM}R_{O_{2}}$ increased by ∼20% at +1.5–2°C (∼10.6%/°C) (see Figure 7), corresponding to a *Q*_10_ of ∼2–3. Even though CBF and $\mathrm{CM}R_{O_{2}}$ are usually connected, during hyperthermia, they are paradoxically uncoupled. Here, CBF is more tightly influenced by reductions in $P_{\mathrm{aC}O_{2}}$, and the maintenance of $\mathrm{CM}R_{O_{2}}$ is accomplished by proportional increases in OEF (reviewed in Bain et al., 2020, 2015b).

### 4.2 Exercise

Exercise in a hot environment, in motivated individuals, can often elevate core temperature (*T*_core_) over 40°C (Lee et al., 2010). Similar to reports at rest (Bain et al., 2020), there are *Q*_10_‐mediated elevations in $\mathrm{CM}R_{O_{2}}$ of ∼7–8% (or 6.1% elevation per 1°C increase in core temperature) (Nybo et al., 2002a) (see Figure 7). Also, as measured via the Kety–Schmidt technique, CBF declined up to 18% during hyperthermic exercise (Nybo et al., 2002a). The authors linked this change to a concomitant 18% lowering of $P_{\mathrm{aC}O_{2}}$, via hyperventilation and thus a hypocapnic‐vasoconstriction‐induced reduction in CBF. In contrast, as measured via Duplex ultrasound, Gibbons et al. (2021) demonstrate a 10% increase in CBF during exercise in heat (+ 2°C in core temperature). These discrepancies between studies are attributed to multiple factors, such differences in exercise intensity and duration, mode of heating, core temperature achieved, magnitude of hypocapnia, and posture. Although dehydration is commonly experienced during exercise in the heat, Trangmar et al. (2015) demonstrated that during incremental cycling to exhaustion in the heat (35°C), despite reductions in CBF and $CD_{O_{2}}$, dehydration (3% of body mass) did not compromise $\mathrm{CM}R_{O_{2}}$ due to compensatory elevations in OEF (Trangmar et al., 2014).

Changes in CBF also function in heat removal from the brain (Shevelev, 1998; Yablonskiy et al., 2000) through the jugular venous blood (Nybo et al., 2002b). However, the change in CBF is not the only way brain cooling can occur. For example, during hyperthermia, excess heat is dissipated through the skull and face as a result of vasodilation in the scalp vessels (Froese & Burton, 1957). This heat removal mechanism, however, was impaired during exercise in hyperthermia compared with normothermia, and heat was continuously stored in the brain (reviewed in Nielsen & Nybo, 2003). Furthermore, during hyperthermic exercise, higher perceived exertion and faster onset of fatigue are imminent. Although the effect of hyperthermia on increasing CNS fatigue (Nybo & Nielsen, 2001) and compromising motor task completion (Piil et al., 2019) has been reported, the exact mechanism underlying such consequences of hyperthermia is poorly explored in humans.

### 4.3 Hypothermia

**FIGURE 8:**
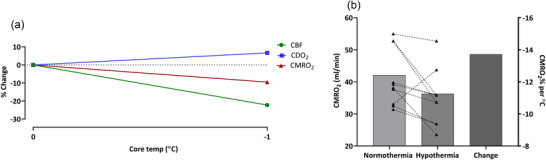
(a) Schematic diagram presenting typical percentage changes in cerebral blood flow (CBF; green), cerebral oxygen delivery ($CD_{O_{2}}$; blue), and the cerebral metabolic rate of oxygen ($\mathrm{CM}R_{O_{2}}$; red) during core cooling of 1.0°C. The core cooling observed in a normoxic environment results in reductions in CBF and $\mathrm{CM}R_{O_{2}}$ in the face of elevations in oxygen delivery. (b) Individual changes in $\mathrm{CM}R_{O_{2}}$ from normothermia to mild hypothermia and the average decline in $\mathrm{CM}R_{O_{2}}$ per °C reduction in *T*_core_. Modified and redrawn from Gibbons et al. (2020) and Howe et al. (2025).

With cold stress and mild hypothermia, a number of systemic responses are activated in an attempt to limit heat loss. To minimize heat loss, peripheral vasoconstriction and piloerection increase the body's physiological shell, while shiver‐induced thermogenesis accelerates heat production (Castellani & Young, 2016; Parsons, 2007; Stocks et al., 2004). These two thermal‐mediated responses also elicit a number of physiological changes that contribute to protection from heat loss, and have a great influence on altering CBF, including peripheral vasoconstriction (increasing MAP), haemoconcentration (impact on $C_{aO_{2}}$), ventilation (impact on $P_{\mathrm{aC}O_{2}}$) and metabolism (*Q*_10_). The core cooling‐induced elevations in MAP and haemoconcentration act to increase CBF and maintain $CD_{O_{2}}$. Furthermore, Gibbons et al. (2020) observed a threefold increase in ventilation (reflecting a combination of hyperpnoea and hyperventilation) with core cooling by a water perfusion suit. As a consequence of high ventilation, $P_{\mathrm{ETC}O_{2}}$ and $P_{\mathrm{aC}O_{2}}$ decrease by 10% and 20%, respectively (Gibbons et al., 2020). These reductions in $P_{\mathrm{aC}O_{2}}$, via hypocapnia‐mediated cerebral vasoconstriction, caused a decrease in CBF by 20–30% with a 15% reduction in $CD_{O_{2}}$ (see Figure 8). As stated in the hyperthermia section, temperature also affects $\mathrm{CM}R_{O_{2}}$ in theoretical proportion to its *Q*_10_ coefficient. Such reductions in $\mathrm{CM}R_{O_{2}}$ may at least partly be explained by the influence of the *Q*_10_ coefficient during hypothermia (Figure 8b).

### 4.4 Cold water immersion

**FIGURE 9:**
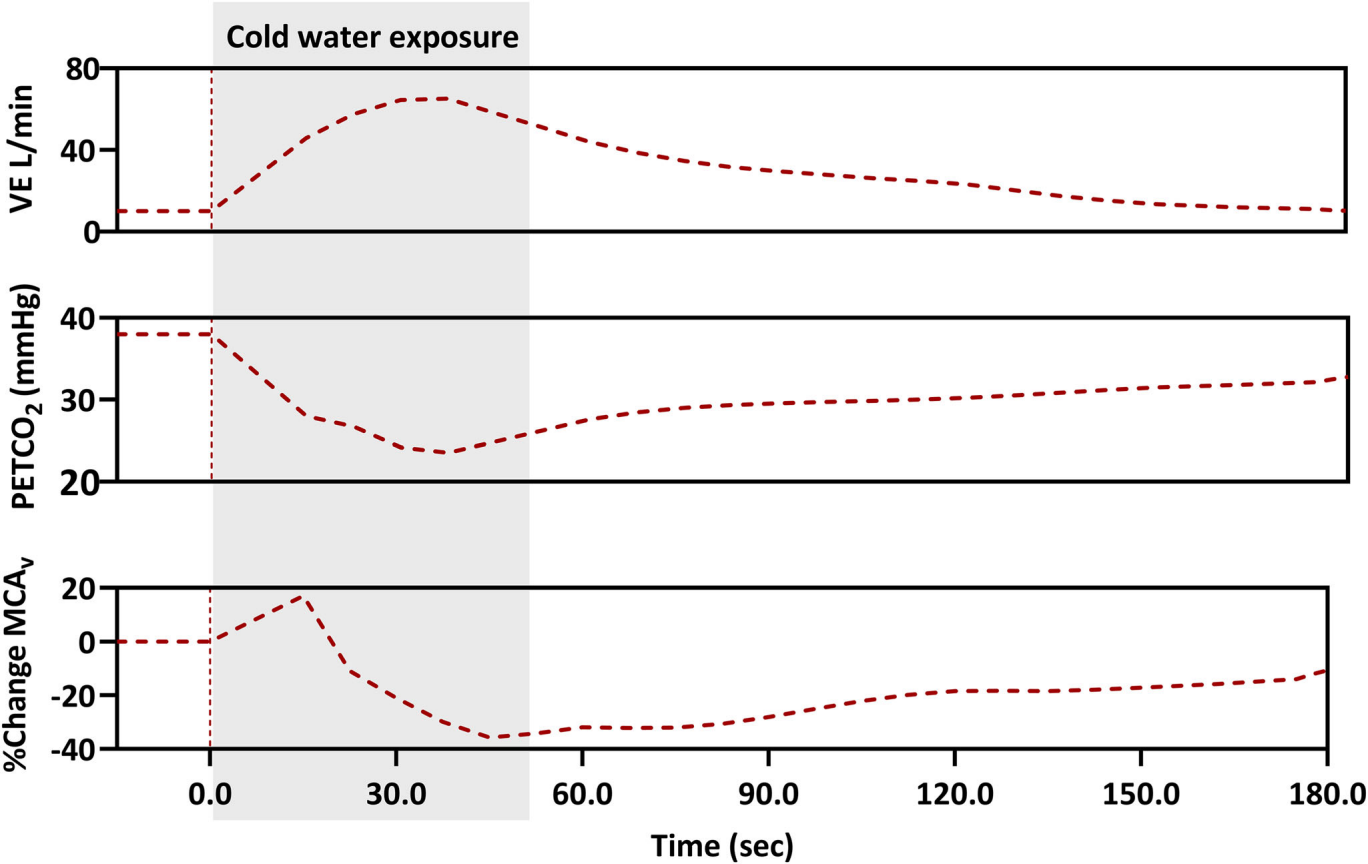
Integrative model of changes to cold water immersion. Sudden cold water immersion induces immediate hyperventilation in the first 30–40 min. Thereafter, hyperventilation reduces the end tidal CO_2_ ($P_{\mathrm{ETC}O_{2}}$), causing cerebral vasoconstriction and reductions in middle cerebral artery velocity (MCAv). Redrawn and modified from Mantoni et al. (2007).

Sudden cold‐water immersion can result in death or serious incapacitation (e.g., loss of motor control or impaired consciousness) of an individual long before frank hypothermia develops (reviewed in Tipton et al., 2022). As such, this response is probably responsible for the majority of annual open‐water immersion deaths. Internationally, there are about half a million immersion‐related deaths each year (Shattock & Tipton, 2012). In relation to the current review, it is well established that initial cold‐water immersion, especially in non‐habituated volunteers, results in rapid hyperventilation‐induced hypocapnia (Cooper et al., 1976). Immediately upon immersion, ventilation increased by 60% with $P_{\mathrm{ETC}O_{2}}$ dropping by a noteworthy 31% in the first 30 s (Mantoni et al., 2007) (Figure 9). Such a hypocapnia response elicits a 10–40% decrease in middle cerebral artery velocity (MCAv) (Mantoni et al., 2008) (reviewed in Datta & Tipton, 2006). The magnitude of hyperventilation and cerebral hypoperfusion appears to be modulated by physical activity upon sudden immersion, i.e., treading water, water temperature, habituation to cold water exposure, and swimming experience (Button et al., 2015).

### 4.5 Exercise

There are limited studies that have measured CBF during exercise in the cold. Upon measuring intracranial velocity via MCAv, Miller et al. (2022) found that changes during cycling exercise in cold environment (5°C) demonstrated no significant difference compared to a normothermic environment (15°C). However, the core temperature revealed no changes comparing cold and thermoneutral environments. Similarly, the authors report that even prolonged exposure to exercise in cold (50 min/day, 3 times/week in 5°C for 8 weeks of intervention) did not elicit differences in CBF. However, a significant improvement in dynamic cerebral autoregulation was observed postintervention when exercising in a cold environment. The authors stated that cold exercise training improved both the magnitude of the MCAv velocity response and temporal alignment to forced blood pressure oscillations. The physiological implications of these findings are unclear and measures of CBF or $\mathrm{CM}R_{O_{2}}$ were not made.

### 4.6 Implications on cerebrovascular function in thermal stress

Heat stress‐related alterations in cerebrovascular functioning range from mild dizziness to pathophysiological heat illness associated with reversible or permanent cognitive impairments to death (reviewed in Hales et al., 1994; Sawka et al., 2011). Even at a mild increase in core temperature, physiological perturbations occur (e.g., cardiovascular strain, respiratory‐induced alkalosis, and metabolic changes) (reviewed in Coyle & González‐Alonso, 2001; Souissi et al., 2021). It should also be noted that cognitive impairments during heat stress appear to also be influenced by hydration status (Nielsen & Nybo, 2003; Nybo et al., 2002b). An increased cerebral temperature can impair blood–brain barrier integrity, particularly when combined with exercise and dehydration (Watson et al., 2006). Hence, developing mitigation techniques and interventions would be invaluable for cerebrovascular coping strategies in response to heat stress. Some studies showed that mild hypothermia has been shown to have neuroprotective effects (Walters et al., 2011); however, phase 2 and 3 clinical trials have revealed that therapeutic hypothermia to reduce intracranial pressure did not improve clinical outcomes (Andrews et al. (2015) and that hypothermia was not associated with improved survival and functional outcome in patients with cardiac arrest and non‐shockable rhythm (Taccone et al., 2024). At least in non‐hospitalized humans, in view of widespread popularity, the benefits of cold‐water immersion or cold plunges on cerebrovascular health warrant future research.

## 5 CEREBRAL BLOOD FLOW REGULATION IN MICROGRAVITY

**FIGURE 10:**
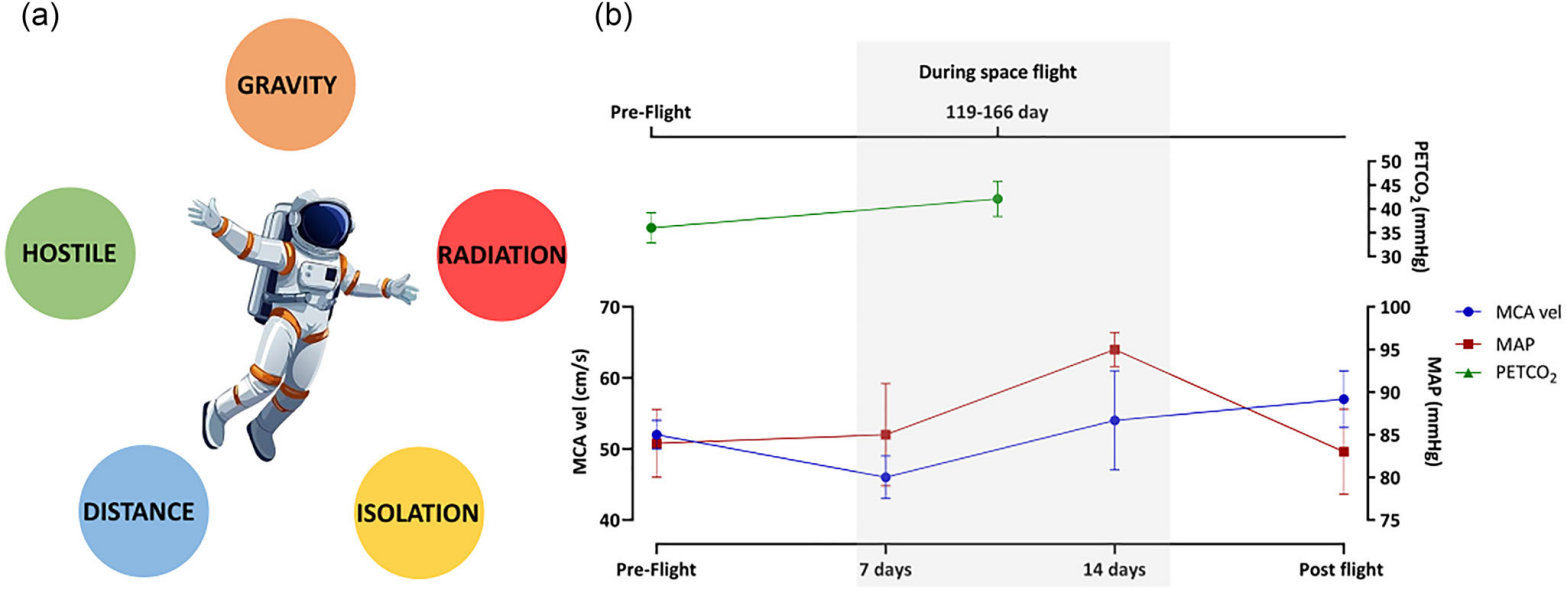
Space exposome and brain strain. (a) The space exposome reflects the totality or cumulative sum of all environmental stressors an astronaut experiences during spaceflight that includes prolonged microgravity. The cerebrovascular response is highly complex and difficult to simulate given the synergies between environmental stressors, multitude of organ systems affected and extraneous factors. (b) Changes in cerebral blood velocity in the middle cerebral artery, accompanied by changes in MAP and $P_{\mathrm{ETC}O_{2}}$ seen during spaceflight. Redrawn and modified from Arbeille et al. (2001, 2021), Bailey (2025), Hughson et al. (2016), Iwasaki et al. (2007).

The altered gravitational gradients experienced during spaceflight – from microgravity in orbit to the hypergravity encountered during launch and re‐entry – pose unique challenges to the astronaut's brain due to the elimination of the hydrostatic gradient that normally governs blood distribution in the human body (Bailey et al., 2025; Goswami et al., 2025). The cumulative sum of all environmental hazards encountered during spaceflight, termed the space ‘exposome’, encompasses altered gravity, prolonged isolation and confinement at increasing distances from Earth, and higher doses of cosmic radiation (Bailey, 2025; Bailey et al., 2025). Precisely how the space exposome will collectively impact cerebrovascular function in deep space is unknown given our poor inability to accurately measure cerebrovascular function as well as simulate multistressor dynamics and determine to what extent stressors exert simple linear additive or complex coupled nonlinear synergistic effects (Figure 10) (Bailey, 2025). In many ways, the space ‘exposome’ provides another example of the influence of coexposures on CBF regulation (see next section).

So far, although a number of elegant studies have been conducting in brief parabolic flights (Klein et al., 2020; Lawley et al., 2017b; Marshall‐Goebel et al., 2024), the main exploration area of cerebrovascular function has been during and after short‐duration spaceflights (Arbeille et al., 2001; Bagian & Hackett, 1991; Blaber et al., 2011; Iwasaki et al., 2007). Only a few measurements have been made during or after long‐duration flights (Arbeille et al., 2001; Tobal et al., 2001). Previous studies reported subtle increases in cerebrovascular resistance (as assessed via TCD and blood pressure) after 3 months in space (Arbeille et al., 2021). Although $P_{\mathrm{aC}O_{2}}$ was not measured, it is hypothesized that these changes reflect increased sympathetic vasoconstriction. Additionally, several studies reported CBF in humans exposed to microgravity. For example, Bagian and Hackett (1991) showed no clear change between pre‐ and in‐flight MCAv measurements. Similarly, Arbeille et al. (2001) reported stable MCAv during space missions lasting 6 days to 6 months. However, Iwasaki et al. (2007) showed an initial decrease in MCAv with a return to normal after 2 weeks in space. Interestingly, significant elevations (+6 mmHg from preflight baseline) in $P_{\mathrm{ETC}O_{2}}$ after 116 days of spaceflight have been reported (Hughson et al., 2016). Such hypercapnia would be expected to have a marked influence on CBF; however, that related elevations in MCAv were not also observed would suggest some acid–base compensation of the respiratory acidosis and/or potentially some limitation of TCD to reflect flow (Ainslie & Hoiland, 2014) during conditions of microgravity.

### 5.1 Implications for cerebrovascular function in microgravity

A cephalic (headward) shift of blood and cerebrospinal fluid (CSF) of up to 2 L is one of the most immediate and pronounced effects of microgravity and contributes to an elevation in central venous pressure, intracranial pressure and cerebral venous congestion (Lawley et al., 2017b; Moore & Thornton, 1987). Corresponding structural changes in space include upward brain displacement, alterations in CSF dynamics, impaired neurovascular unit integrity and increased perivascular space morphology (Bailey et al., 2020; Goswami et al., 2025). Neuroimaging reveals ventricular expansion (7–25%), particularly around the ventral frontal, temporal and occipital lobes, CSF redistribution, and grey/white matter compression near the brain's vertex (Koppelmans et al., 2016; McGregor et al., 2023; Seidler et al., 2024). These features parallel clinical conditions such as intracranial hypertension and normal pressure hydrocephalus (Seidler et al., 2024). Potential mechanisms include upward brain shift leading to sagittal sinus compression, and corresponding impairment in glymphatic clearance (Seidler et al., 2024). Furthermore, spaceflight may lead to phenomena not previously observed in ground‐based or weightlessness studies, such as potential retrograde venous flow from central veins through the internal jugular veins that can predispose to focal thrombosis (Fall & Bailey, 2025; Marshall‐Goebel et al., 2019). These fluid shifts, along with potential acidosis (Hughson et al., 2016), pose a major risk to mission operational success by contributing to headaches, malaise, cognitive impairment and a constellation of adverse changes in visual acuity known collectively as the spaceflight‐associated neuro‐ocular syndrome or SANS (Mader et al., 2011). To a much lesser extent, even at sea level in the absence of microgravity, posture can have a marked influence on CBF (large via changes in $P_{\mathrm{aC}O_{2}}$) and on cerebral venous outflow (i.e., the internal jugular vein is known to collapse in the upright posture whereas the cerebral veins and dural sinuses do not (Gisolf et al., 2004).

## 6 CONSEQUENCES OF CO‐EXPOSURES OR CROSS‐ACCLIMATIZATION FOR CBF REGULATION

As reviewed herein, with the exception of cold and gravity, the human cerebrovascular consequences of varying forms of hypoxia and heat stress have been relatively well studied. It is noteworthy, however, that the vast majority of these studies have almost always focused on the impact of single stressors on the brain. However, coexposure to environmental stressors better represents real‐world environments. Such influence on CBF is selectively challenging more different physiological responses simultaneously, which can impact brain function (i.e., its oxygenation, temperature, pressure, etc.). Also, coexposure presents a higher challenge for cerebral homeostasis; hence, some responses are prioritized when the brain approaches physiological limits. In spite of the importance of these real‐world conditions, the investigation of human physiological responses to even one stimulus, such as heat, cold or hypoxia, is challenging. Nevertheless, while studies that examine human responses to combined stresses are more realistic (e.g., hypoxia and cold), their interpretation is complex given the number of coreflexive and interactive physiological systems involved (see Figure 10 for example). Therefore, there are still relatively few of these studies, especially with a focus on cerebrovascular function. Nevertheless, those that have been done offer important insights into an exciting and emerging area within human physiology: cross‐acclimation and cross‐tolerance. Cross‐acclimation (adjustments derived from exposure to a simulated environment) (Horowitz, 2007) and cross‐acclimatization (adjustments derived from exposure to a natural environment) (Salgado et al., 2014) usually define physiological changes that are short‐lived and not phenotypically inherent. Furthermore, cross‐tolerance has been suggested as a term to describe cellular and molecular pathways relevant to adaptations between thermal and hypoxic environments; as will be discussed, this may or may not result in improved tolerance and/or habituation (reviewed in Ely et al., 2014). Elegant reviews are available on this general topic (Gibson et al., 2017; Lee et al., 2019; Tipton, 2016; Willmott et al., 2024), and herein we summarize some of the limited and selected studies that have investigated the impact of coexposures in the context of high altitude and hyperthermia on CBF regulation.

### 6.1 Altitude‐induced hypothermia

**FIGURE 11:**
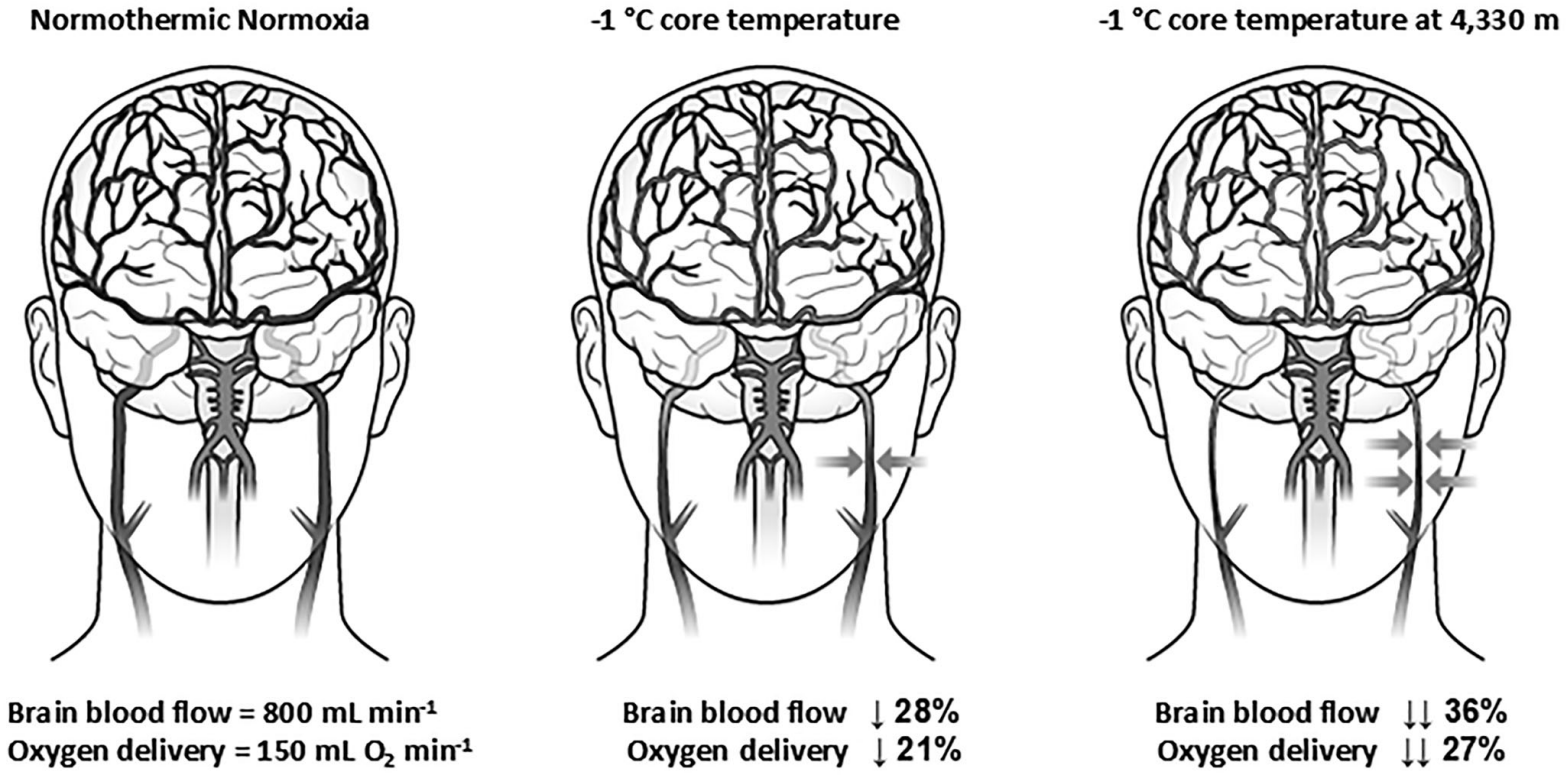
The influence of mild hypothermia at sea level and following acclimatization to high altitude. Note the additional compromise in brain blood flow and oxygen delivery when hypothermia occurs at high altitude. These findings highlight the fact that the interaction of cold and hypobaric hypoxia poses a much greater threat to the brain than either stressor in isolation (Cheung & Ainslie, 2022; Gibbons et al., 2022).

Upon ascent and following prolonged stay at high altitude, apart from hypoxia, trekkers and climbers are often exposed to thermal stress (i.e., hypothermia). The evidence of these stressors combined is sparse and lacking. In one of the first studies of its kind, the regulation of oxygen delivery to the brain was investigated under moderate cold stress at sea level (344 m) and after 16 days residing at altitude in Cerro de Pasco, Peru at 4330 m (Gibbons et al., 2020). Core cooling (induced by immersion in cold water) by 1°C at both altitudes caused hyperventilation and a subsequent 5–8 mmHg decrease in $P_{\mathrm{aC}O_{2}}$, which contributed to decreasing CBF (shown in Figure 11). At sea level, the cold stress decreased CBF by 28% and brain oxygen delivery by 21%. At high altitude, while $CD_{O_{2}}$ in the normothermic brain was already 11% lower than sea level, oxygen delivery was reduced by an additional 18% with core cooling. This resulted in a $CD_{O_{2}}$ to the brain that was 27% lower than at normothermic body temperature at sea level. These findings highlight the fact that the interaction of cold and hypobaric hypoxia poses a much greater threat to the brain than either stressor in isolation. Additionally, the same study showed the highest [Hb] and $C_{aO_{2}}$ when core cooling at high altitude was elicited. Since haemoconcentration with core cooling is consequent to the combined factors of cold‐induced diuresis (Pozos & Danzl, 2001), splenic contraction (Baković et al., 2005; Kanter, 1968), and plasma leakage from the vascular space (Wolf et al., 1992), interactive regulation of CBF and $CD_{O_{2}}$ with these factors would provide more knowledge on the topic of combined stressors. However, this area remains largely unexplored.

## 7 FUTURE RESEARCH DIRECTIONS

So far in this review, the main environmental stressors and their influence on CBF and its metabolism have been compared and contrasted. However, many questions remain unanswered and some selected unexplored areas are highlighted briefly next.

### 7.1 Most studies in young healthy males

Historically, due to social and sex biases, there has been disproportionate physiology‐based research targeting males versus females (Usselman et al., 2024). According to Avery and Clark (2016), sex‐related reporting in randomized controlled trials leads to limited insights into female physiology and inadequate translation into the additional impact of environmental stress. This is noteworthy giving evidence that females show significantly higher (up to 40%) resting CBF compared to males (Muer et al., 2024), with differences throughout the menstrual cycle (Peltonen et al., 2016) and with menopause (reviewed in Ruediger et al., 2021). Further evidence in animal models support a key role of oestrogen in both resting CBF and reactivity (Deer & Stallone, 2016; Geary et al., 2000; Skarsgard et al., 1997). Similarly, in addition to thermoregulation capacity, there are marked changes in CBF and metabolism in vulnerable populations such as children and the elderly. Clearly, the influence of sex, sex hormones, and the interaction with age and environmental stressors on CBF regulation remains to be elucidated.

### 7.2 Co‐exposures influence on CBF regulation

Effects of environmental stressors such as heat or hypoxia are relatively well studied. However, when observing situations in which they occur (e.g., high‐altitude, exercise, apnoea), it could be seen that the interplay or coexposure of environmental stressors is present (see Figures 1, 10 and 11) yet poorly studied. As outlined earlier, two main common coexposure situations stand out. First, heat and hypoxia coexist externally in many places in the world, especially on high plateaus [e.g., southwestern USA (Colorado Plateau), Africa (Simien Plateau) and at equatorial mountain ranges (Andes; southern Rockies)]. For example, summer temperatures in the valley of Arizona (Phoenix, Tempe) reach 40–50°C, whereas on the Colorado plateau they are ∼10°C lower, yet still reach 33°C in Flagstaff at 2300 m. The impact of such heat and hypoxia on cerebrovasuclar function and health remains to be explored. Second, during breath‐hold diving, divers experience the diving mammalian reflex (Andersson & Schagatay, 1998; Andersson et al., 2004; Lindholm & Lundgren, 2006). The so‐called diving mammalian reflex is primarily connected to cold water immersion or face cooling, which induces physiological body responses such as bradycardia, sympathetically mediated splenic and peripheral vasoconstriction (reviewed in Schagatay, 2009; Baković et al., 2005; Heusser et al., 2009). As stated in the above sections, the *Q*_10_ coefficient of biological tissue could reduce metabolism by ∼6–8% per °C. Therefore, the thermal mechanism for reductions in $\mathrm{CM}R_{O_{2}}$ during a dry static breath hold in humans might be relevant when a breath hold is performed in cold water. Hence, reducing the temperature of the core may play a crucial role in preserving oxygen. When observing other diving marine mammals, seals are able to reduce their body core and brain temperature by ∼2.5°C during a 20‐min breath hold in 4°C water (Blix et al., 2010). Therefore, it could be assumed that such ‘external stress and internal strain’ would be beneficial for breath‐hold divers preserving oxygen. However, there is a possibility that cooling affects motor control and diminishes swimming performance, which could negatively affect divers (Tipton et al., 1999), but further research is needed.

### 7.3 Other environmental stressors in a changing climate (space, humidity, UV radiation and pollution)

Although this review focuses on well‐studied environmental stressors on CBF and $\mathrm{CM}R_{O_{2}}$, other possible and rising stressors impact CBF regulation and merit future study. As already noted, the space ‘exposome’ provides another example of the influence of co‐exposures via myriad factors that directly or indirectly influence CBF regulation (e.g., microgravity, isolation, stress, radiation). One of the concerning increases in modern society is atmospheric pollution coming from increased wildfire appearances. Such conditions, which combine extreme heat and smoke‐associated air pollution, could induce neuroinflammation and compromise cognitive function and blood–brain barrier integrity (reviewed in White, 2024). Additionally, many other factors may induce air pollution and have adverse effects on the brain, for example, pollution from transportation, industrial activities, agriculture and household combustion. However, as far as we are aware, no studies have examined the influence of air pollution on CBF and cerebral regulation. Other environmental stressors, such as humidity and UV radiation, pose additional challenges to CBF control that are largely unexplored, but seemingly have considerable neurocognitive effects (Piil et al., 2020). Future studies are needed to assess the myriad multistressor exposures, to determine the influence on not only CBF and metabolism but also their effects on blood–brain barrier integrity, synaptic signalling and cognition more realistically. In the context of climate change, not only are environmental extremes becoming more extreme, but more and more people are being exposed to these extremes across the world.

### 7.4 CNS omic approaches to understand the cellular consequences of environmental exposures

So far influence of environmental stressors on the human brain has been mainly studied by investigating CBF and accompanying metrics (e.g., $\mathrm{CM}R_{O_{2}}$, $CD_{O_{2}}$, ICP). Omic‐based approaches (Owens & Bennett, 2024) combined with cross‐brain sampling could serve as new indicators of molecular strain that is placed upon the CNS. Technologies such as transcriptomics, proteomics and metabolomics as the three major omics fields that provide different types of biological information may provide a new avenue for probing new metabolomic and proteomic signalling pathways targeted to the CNS in response to different environments.

## 8 CONCLUSION

Throughout the previous years and decades, multiple studies have examined the influence of different environmental stressors on cerebrovascular regulation. With this review, we have attempted to compare and contrast these various stresses and their impact on CBF and metabolism. When comparing all of the stressors that may impact CBF, alveolar ventilation‐induced changes in $P_{\mathrm{aC}O_{2}}$ seem to be the most important. For example, during prolonged apnoea, elevations in $P_{\mathrm{aC}O_{2}}$ seem to mediate reductions in $\mathrm{CM}R_{O_{2}}$. Similarly, during both hyper‐ and hypothermia, the reduction in $P_{\mathrm{aC}O_{2}}$ acts to determine the related changes in CBF, despite directionally opposite elevations in MAP (with hypothermia) or $\mathrm{CM}R_{O_{2}}$ (with hyperthermia). Even in space the reported elevation in $P_{\mathrm{ETC}O_{2}}$ of 6 mmHg (Hughson et al., 2016) would seem not to interfere with CBF, since MCAv is relatively stable throughout a spaceflight (Arbeille et al., 2001; Iwasaki et al., 2007). During exposure to acute poikilocapnic hypoxia, the magnitude of the hypoxic ventilatory response determines the prevailing $P_{\mathrm{aC}O_{2}}$ and degree of cerebral vasodilation (Ainslie & Poulin, 2004). In contrast, however, changes in $P_{\mathrm{aC}O_{2}}$ may play an intimate role in the regulation of CBF during ascent and acclimatization to altitude. Understanding how the brain responds, adapts to and tolerates these real‐world environmental stressors will inform preventative practices to manage this burden on the brain. Perhaps more importantly, this information will enlighten the research (and broader) community about the real consequences of environmental stress and climate change for human brain function and survival.

## AUTHOR CONTRIBUTIONS

Vrdoljak Dario and Philip N. Ainslie conceived and designed the review. Vrdoljak Dario, Philip N. Ainslie, and Gibbons D. Travis drafted the review. Vrdoljak Dario prepared the figures. Vrdoljak Dario, Gibbons D. Travis, Philip N. Ainslie, and Bailey M. Damian revised and edited the review. All authors approved the final version of the review and agree to be accountable for all aspects of the work in ensuring that questions related to the accuracy or integrity of any part of the work are appropriately investigated and resolved. All persons designated as authors qualify for authorship, and all those who qualify for authorship are listed.

## CONFLICT OF INTEREST

None declared.
